# Development of tea-based (*Camellia sinensis* L.) beads containing probiotics to enhance survival during the gastrointestinal simulation process

**DOI:** 10.1007/s10068-026-02173-1

**Published:** 2026-05-12

**Authors:** Alejandra Lazcano-Armenta, Carolina Ramírez-López, Diana Milena Torres-Cifuentes, Irving Israel Ruiz-López, Carlos Enrique Ochoa-Velasco, Paola Hernández-Carranza

**Affiliations:** 1 Facultad de Ciencias Químicas, Benemérita Universidad Autónoma de Puebla, 72000 Puebla, Mexico; 2 Centro de Investigación en Biotecnología Aplicada, Instituto Politécnico Nacional, Santa Inés Tecuexcomac-Tepetitla, Km 1.5, Tepetitla de Lardizábal C.P. 90700, Tlaxcala, Mexico; 3 Facultad de Ingeniería Química, Benemérita Universidad Autónoma de Puebla, 72000 Puebla, Mexico

**Keywords:** Gastrointestinal fluids, Prebiotics, Antioxidants, Human health

## Abstract

Tea (*Camellia sinensis* L.) has phenolic compounds that have been defined as prebiotics capable of improving probiotic survival in the human gut. This study aimed to use probiotic-containing tea to produce sodium alginate beads to enhance probiotic survival during gastrointestinal simulation (GS). Teas with different oxidation levels (black, green, and white) were evaluated for total flavonoids, total phenolic compounds, antioxidant capacity, and probiotic survival (*Lactiplantibacillus plantarum* and *Lacticaseibacillus casei*) for 4 days of storage. White tea and *L. plantarum* were selected for bead production and GS because white tea showed the highest bioactive compound content and antioxidant capacity, while *L. plantarum* showed better survival during storage than *L. casei*. Although the tea-based beads improved probiotic survival during GS, most viable cells remained retained within the beads (5.66 ± 0.01 log10 CFU/g), while fewer cells were released into the simulated gastrointestinal fluids (4.28 ± 0.20 log10 CFU/g; p < 0.05).

## Introduction

Tea (*Camellia sinensis* L.) is one of the most popular beverages, consumed by over 3 billion people worldwide. It originated in China, where it has spread globally (Zhao et al., 2022). Tea can be classified by degree of oxidation: white tea is neither oxidized nor fermented, and black tea is the most processed. These processes change their chemical composition, sensory attributes, and bioactive compounds (Aloo et al., 2024). In general, tea contains phenolic compounds (catechins and flavonol glycosides as the most important), caffeine, theanine, polysaccharides, and volatile organic substances (Samanta, 2022). Many of these compounds display anti-inflammatory, antimutagenic, antipathogenic, and antiviral properties, which are associated with the prevention of liver, cardiovascular, and neurodegenerative diseases, as well as type 2 diabetes and some cancers (Singh et al., 2024).

Recent studies have indicated that tea is a good source of prebiotics due to its polysaccharide content and high phenolic compound content (Hutkins et al., 2025). Specifically, polysaccharides found in tea flowers can resist digestion in the upper gastrointestinal tract and be metabolized by the gut microbiota. Additionally, the phenolic compounds in tea flowers exhibit strong scavenging activity against reactive oxygen and nitrogen species (Chen et al., 2024; Rojas-Rejón et al., 2024). In this sense, the antioxidant properties of tea have been extensively studied; however, research focusing on its role as a prebiotic to promote probiotic growth remains limited.

Probiotics are defined as living microorganisms that can improve human health when consumed in adequate amounts (more than 9 log10 CFU per day). To provide their benefits, enough viable probiotic cells must reach specific sites in the gastrointestinal tract (Heidarrezaei et al., 2025). Various techniques, such as bead production, encapsulation, lyophilization, and the application of edible films and coatings, have been used to enhance the viability and tolerance to sublethal gastrointestinal stress and storage conditions. Among these methods, bead production using sodium alginate is one of the most popular ways to protect probiotics from environmental factors. However, the use of tea as a prebiotic source in combination with sodium alginate for protecting probiotics has been scarcely studied. For example, Vodnar and Socaciu (2014) indicated that selenium-enriched green tea microencapsulated with chitosan-coated alginate improved the survival of probiotics *L. casei* and *L. plantarum* in the gastrointestinal system. Recently, the use of dark tea polysaccharides combined with reinforced alginate matrix enhanced the viability of *L. acidophilus* during gastrointestinal simulation and storage at room temperature (Fu et al., 2023). However, studies have quantified the viability of probiotics within the bead, considering that it is degraded by gastrointestinal fluids. Nevertheless, the possibility that the beads do not break down during their passage through the gastrointestinal tract has not yet been explored. This means they could pass through the digestive system intact, potentially releasing only the probiotics attached to their surface.

Therefore, this study aimed to develop tea-sodium alginate beads containing probiotics to enhance their survival during gastrointestinal simulation. To achieve this objective, the following topics were covered: (i) evaluation of the bioactive compounds and antioxidant capacity of black, green, and white teas, as well as the survival of inoculated probiotics (*L. plantarum* and *L. casei*) during storage, (ii) selection of the sodium alginate concentration to formulate tea-based beads inoculated with selected probiotic to evaluate their release in the gastrointestinal fluids, and (iii) assess the survival of probiotics in different parts of the beads (interior and surface) and gastrointestinal fluids.

## Materials and methods

### Probiotic microorganisms

*Lactiplantibacillus plantarum* (NRRL B-4496) and *Lacticaseibacillus casei* (NRRL B-1922) were obtained from the microbial collection of the Faculty of Chemical Sciences of the Benemerita Universidad Autonoma de Puebla. For reactivation, the microorganisms were thawed (20 ± 3 °C) and cultured at 37 ± 2 °C in Man-Rogosa-Sharpe (MRS) broth for 24 h (early stationary phase, 1–10 × 10^9^ CFU/mL). The probiotics were immediately used to inoculate various sterilized teas.

## Tea preparation and probiotic inoculation

**Fig. 1:**
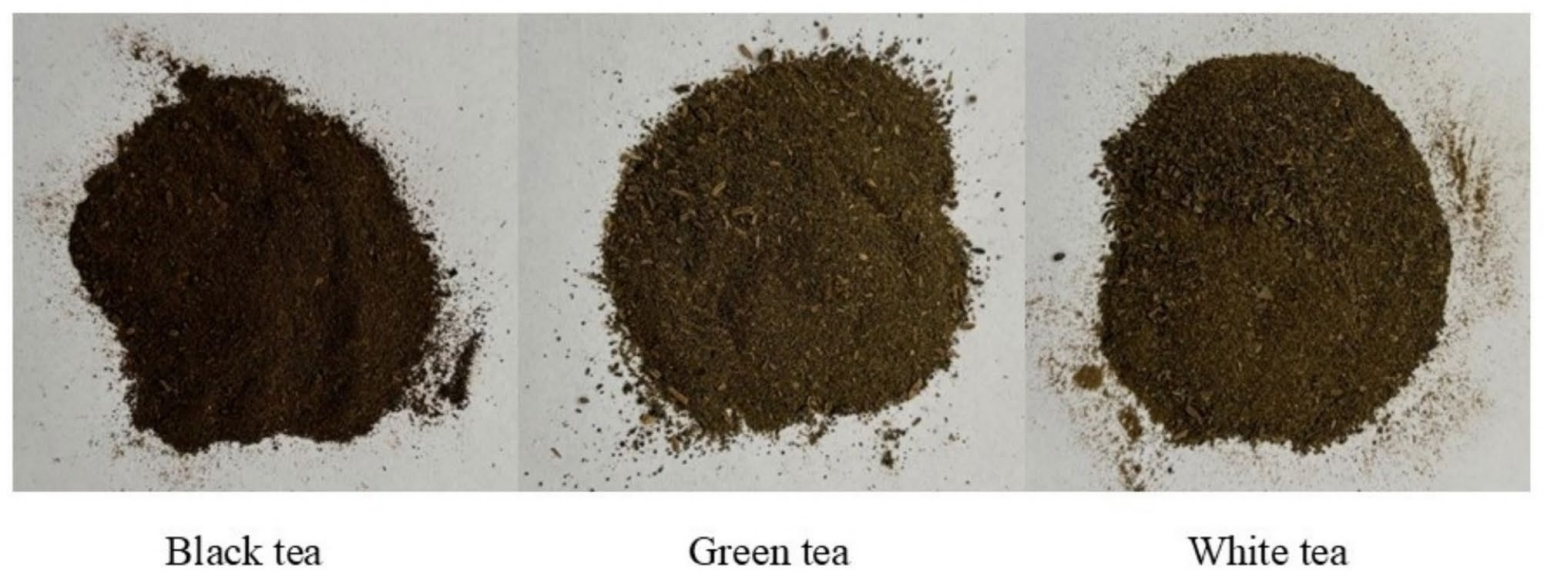
Black, green, and white teas used in this study

Black, green, and white teas (*Camellia sinensis* L.) were acquired from Molienda Sagrada, a Tea house, which exported them from the Region of Anhui, China (Fig. 1). Tea leaves with a moisture content in the range of 5.89—8.69% (oven-drying at 105 ± 5 °C, FE-292 model, FELISA, Zapopan, Jalisco, Mexico) were ground and sieved to obtain a powder (180 µm). Tea infusions were obtained by placing 0.1 g of tea powder in 100 mL of drinking water. To evaluate the effect of temperature on the bioactive compounds and antioxidant capacity of tea, the extraction was conducted by agitation (230—240 rpm) at two temperatures, 20 ± 2 °C and 80 ± 3 °C for 1 h and 5 min, respectively. Then, to remove tea particles, infusions were filtered through a Whatman # 4 filter and used immediately for analysis. The teas with the highest bioactive compounds and antioxidant capacity were selected to be sterilized at 121 °C for 15 min and subsequently inoculated with 100 µL of either *L. plantarum* or *L. casei* in the early stationary phase (1—10 × 10^9^ CFU/mL) to achieve an initial probiotic concentration in the range of 5.00 to 6.00 log10 CFU per mL. Then, the teas were incubated at room temperature (20 ± 3 °C) for further analysis of their bioactive compounds, antioxidant capacity, and probiotic survival.

## Bioactive compounds and antioxidant capacity quantification

The teas were analyzed in their total flavonoids (TF, mg catechin/100 mL), total phenolic compounds (TPC, mg GAE/100 mL), and antioxidant capacity (AC, mg Trolox/100 mL) using the methodology proposed by Hernández-Carranza et al. (2016). For TF, catechin was used as a standard. Whereas gallic acid and Trolox were used as standards for TPC and AC, respectively.

## Effect of tea on the probiotic survival during storage

The survival of probiotics in teas during a 4-day storage at room temperature was evaluated by serial dilution of tea with peptone water until 30—300 CFU were counted. The sample was plated in MRS agar and cultured at 37 ± 2 °C for 24 h under anaerobic conditions. The probiotic count was reported on a logarithmic scale (log10 CFU/g). The tea with the highest antioxidant properties and probiotic survival was selected for the bead process and subsequent analysis under gastrointestinal simulation.

## Production of alginate beads of selected tea containing probiotics

Selected tea and the probiotic microorganism were used to produce beads to evaluate the effect of gastrointestinal simulation (GS) on probiotic survival. Ionic gelation with sodium alginate (SA, 0.75% and 3.00% w/v) and calcium chloride solution (0.1 M) was used for the bead production. To produce beads, the tea inoculated as previously described was used for dissolving 0.75 or 3.00 g of SA. Finally, solutions were syringe-dropped into a calcium chloride solution to produce the beads with an average diameter, area, and volume of 4.83 ± 0.38 mm, 82.92 ± 10.91 mm^2^, and 93.86 ± 13.05 mm^3^, respectively. The diameter of the beads (*n* = 10) was determined using a micrometer with a sensitivity of 0.01 mm (IP54, Qfun, China), whereas area (*A*) and volume (*V*) were calculated as $A=4\pi {r}^{2}$ and $V=\frac{4}{3}\pi {r}^{3}$, respectively. Where* r* is the radius of the sphere, and π is the well-known relation between diameter and circumference of the sphere.

## Analysis of beads under gastrointestinal simulation

**Fig. 2:**
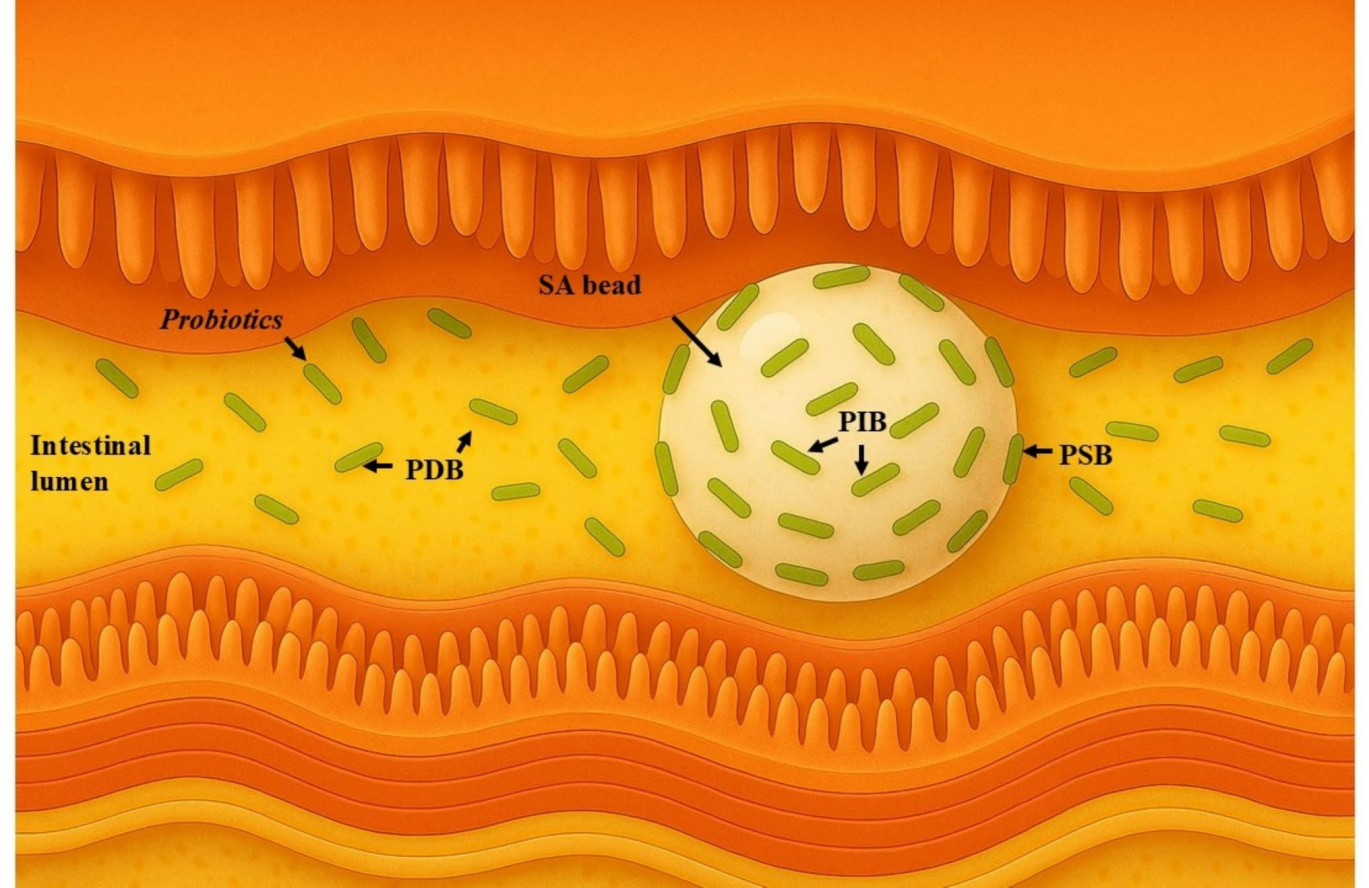
Schematic representation of probiotics in white tea beads in the gastrointestinal system. PIB: probiotic inside the bead. PSB: probiotic on the surface of the bead. PDB: probiotic detached from the bead. SA: Sodium alginate

To evaluate the effect of GS on the survival of the probiotic in SA beads and its surrounding environments (Fig. 2), the following experiments were performed. C: free probiotics (without bead), PIB: probiotics inside the bead, PSB: probiotics on the surface of the bead, PWB: probiotics in the whole bead (PIB plus PSB), PDB: probiotics detached from the bead, PDIBG: probiotics detached from the intact bead into the gastric fluid, PDFBG: probiotics detached from the fractured bead into the gastric fluid, PDIBI: probiotics detached from the intact bead into the intestinal fluid, PDFBI: probiotics detached from the fractured bead into the intestinal fluid. Probiotics obtained from the different parts of the bead and its surroundings were immediately quantified as previously described.

## Survival of probiotics during gastrointestinal simulation

### Probiotic survival by plating assay

The survival of probiotics during the gastrointestinal simulation process was evaluated following the methodology proposed by He et al. (2021) with some modifications. To know the impact of mastication on probiotic survival during GS, beads were fractured using a manual press. One bead was placed under the manual press and allowed to impact with its own weight. The measurement of the impact was measured with a pinch gauge dynamometer (Saehan Corporation, Korea), and the fractured force (7.23 ± 0.10 kg/cm^2^) was calculated (*n* = 10) as the relation between the force and the area of the bead. Gastric and intestinal solutions were used to simulate the gastrointestinal system in 1:1 ratio of beads and digestive fluids. This relation was selected due to guarantee wetting and lubrication of the meal in the gastrointestinal system (Dixit et al., 2024). The gastric solution was formulated by diluting pepsin (3.2 g) and NaCl (2 g) in 1 L of sterile distilled water. The pH was adjusted to 2.0 using a HCl (1 M) solution. On the other hand, the intestinal solution was prepared by diluting 10 g of pancreatin and 6.8 g of K_2_HPO_4_ in 1 L of sterile distilled water; pH was set to 7.0 with NaOH. Then, 5 mL of selected tea (control) or 5 g of SA beads were placed with 5 mL of gastric solution for 2 h at 110 rpm and 37 °C. Then, 5 mL of the gastric solution containing tea or 5 g of SA beads was taken and placed in 5 mL of intestinal solution for 3 h at 110 rpm, and 37 °C. The probiotic count was analyzed in the whole bead (PWB) by grinding the beads with a mortar. One gram of the ground was mixed with 9 mL of peptone water. For the surface of the bead (PSB), 1 g of bead was placed with 9 mL of peptone water and allowed to release probiotics for 1 min. To analyze the interior of the bead (PIB), the same bead obtained from the surface test was used. The bead was ground and mixed (1 g) with 9 mL of peptone water. For probiotics released after the gastrointestinal simulation (PDB), fluids from these processes were collected, and 1 mL of the fluids was combined with 9 mL of peptone water. All samples (1 mL each) were serially diluted in 9 mL of peptone water to achieve a count of 30—300 CFU. The samples were then plated on MRS agar and incubated at 37 ± 2 °C for 24 h under anaerobic conditions.

### Probiotic survival by MTT assay

Probiotic survival was assessed using an MTT assay following the methodology proposed by Torres-Cifuentes et al. (2021) with some modifications. MTT assay is a colorimetric test where viable cells can change the MTT reagent (3-(4,5-dimethylthiazol-2—yl)-2,5-diphenyltetrazolium bromide) from yellow to purple color. The purple color intensity is related to viable cells. In brief, 100 µL of gastric or intestinal fluid containing detached probiotics from intact (PDIBG and PDIBI) and fractured (PDFBG and PDFBI) beads were incubated at 30 ± 2 °C for 18 h. Then, 100 µL of MTT (0.5 µg/mL) reagent were added to the mixture. The reaction was left to stand for 4 h at 37 °C. Then, 100 µL of SDS (10% in HCl solution at 0.001 N) were added and allowed to stand for 1 h at 37 °C. The reaction was read at 540 nm using a spectrophotometer (Labsystem Multiskan MS Type 352). Absorbances of gastric and intestinal fluids without probiotic cells were used as controls.

## Statistical analysis

All experiments (biological replicates) were conducted in triplicate (n = 3), and each analysis (technical replicates) was performed in triplicate. The results were analyzed using a comparison of means (α = 0.05) through analysis of variance (ANOVA) using Tukey´s test in Minitab 15 software (Minitab Inc., State College, USA).

## Results and discussion

### Effect of extraction process and sterilization on the bioactive compounds and antioxidant capacity of teas (*Camellia sinensis* L.)

**Fig. 3:**
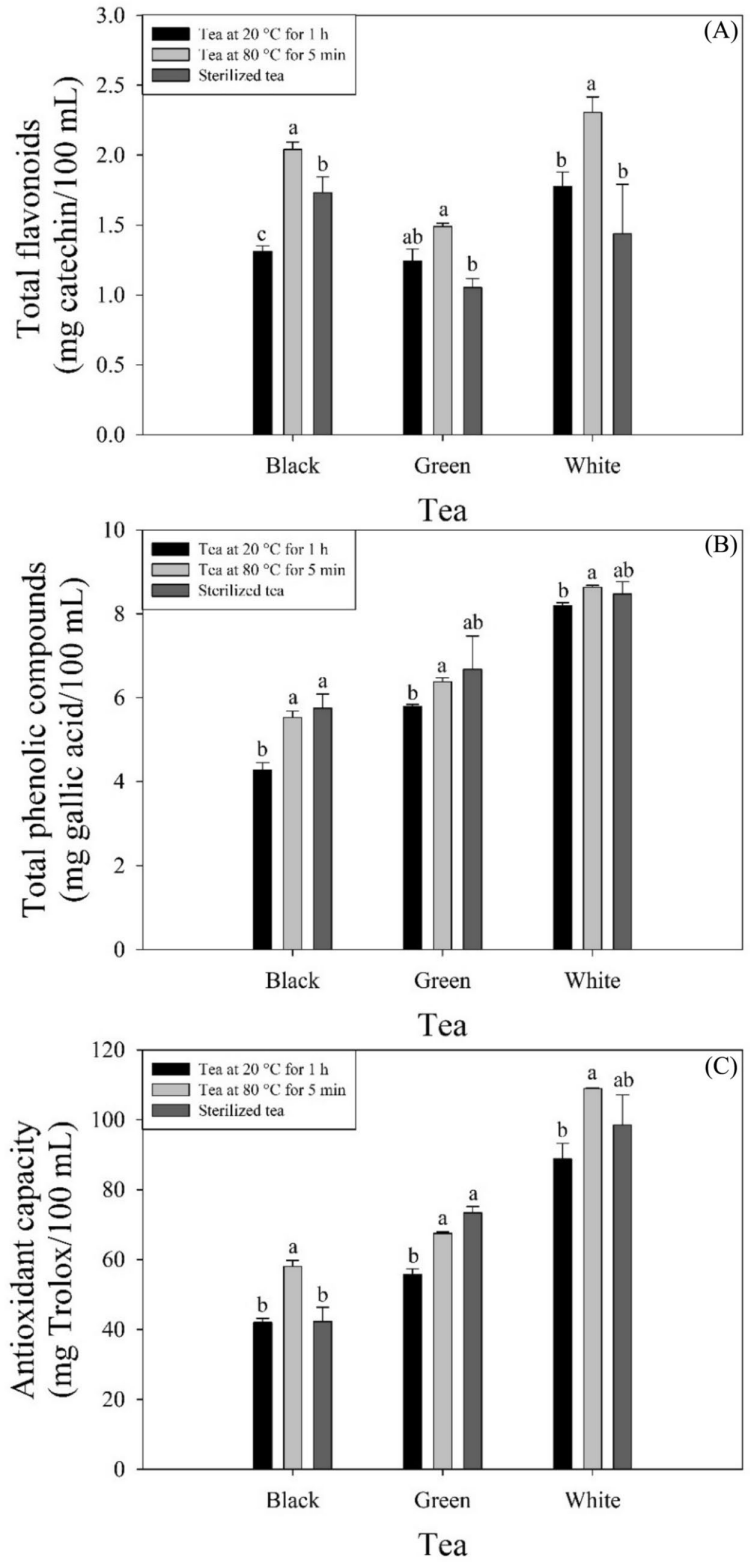
Effect of extraction conditions and sterilization process on the total flavonoids (**A**), total phenolic compounds (**B**), and antioxidant capacity (**C**) of teas (*Camellia sinensis* L.) obtained at 20 ± 2 °C for 1 h and 80 ± 3 °C for 5 min. Different letters indicate statistical differences (*p* < 0.05). Bars indicate standard deviation

Figure 3 shows the effect of the extraction procedure and sterilization process on the TF (A), TPC (B), and AC (C) of white, green, and black teas. As observed, at 20 °C for 1 h, white tea shows the highest TF (1.77 ± 0.10 mg catechin/100 mL), TPC (8.19 ± 0.29 mg GAE/100 mL), and AC (88.80 ± 4.46 mg Trolox/100 mL). In contrast, black tea has the lowest values (1.31 ± 0.04 mg catechin/100 mL, 4.27 ± 0.18 mg GAE/100 mL, 42.01 ± 1.16 mg Trolox/100 mL) for the same bioactive compounds and AC. Some studies have indicated that increasing the processing of tea, such as withering, heat treatment, rolling, fermentation, and drying, can affect bioactive compounds and antioxidant capacity (Aaqil et al., 2023). Specifically, TPC, the main responsible for the AC of tea, ranges from 32.5—75.7 mg/g in white tea, while green tea and black tea contain 12.36—252.65 and 11.33—101.29 mg/g, respectively. Moreover, amino acids such as L-theanine, which also contribute to the AC, are widely present in white tea (7.53—11.91 mg/g) compared to green and black teas with values of 2.63—14.23 and 1.43—11.00 mg/g, respectively. It is interesting to note that both phenolic compounds and L-theanine present lower variability in white tea, which reinforces the effect of processing on the bioactive compounds and AC of tea (Gonçalves Bortolini et al., 2021; Zhou et al., 2022).

In the same Figure, it can be observed that teas obtained at 80 ± 3 °C for 5 min showed the highest bioactive compounds and AC (p < 0.05). The improvements ranged from 20.01% to 55.47% for TF, 5.37% to 29.33% for TPC, and 21.33% to 38.11% for AC. This indicates that using hot water not only reduced brewing time but also enhanced the extraction of bioactive compounds (Ochoa-Velasco et al., 2019). Therefore, teas obtained at the highest temperature were sterilized to evaluate the effect of this treatment on bioactive compounds and AC. It is important to highlight that the sterilization process is commonly used to avoid microbiological spoilage. Results indicated that the sterilization process significantly affected TF. However, this treatment did not affect the TPC and AC of the teas. Interestingly, even after the sterilization process, the bioactive compounds and AC were like those obtained without thermal extraction. Based on these results, teas obtained at 80 ± 3 °C (5 min), plus the sterilization process, were used to evaluate the effect of probiotic growth.

## Probiotic survival, bioactive compounds, and antioxidant capacity of teas during storage

The probiotics survival (*L. plantarum* and *L. casei*) inoculated in teas, as well as their bioactive compounds and AC stability during storage, are shown in Table 1. As observed, during storage at room temperature, *L. casei* population significantly decreased at the end of storage, reaching values below 5.0 log10 CFU/g, regardless of the tea used. However, *L. plantarum* can survive in all inoculated teas (p < 0.05), with values in the range of 6.10—6.13 log10 CFU/g. In this regard, *L. plantarum* possesses the necessary enzymes (glycosidases, esterases, reductases, and decarboxylases) to metabolize the hydroxybenzoic and hydroxycinnamic acids from their natural esterified forms (Landete et al., 2021). During this process, *L. plantarum* can hydrolyze sugars, such as glucose, from glycosidic forms of phenolic acids and flavonoids, using them as a nutrient source for survival during storage (Muñoz et al., 2024). This capacity explains the enhancement (p < 0.05) of TF during storage in all teas containing *L. plantarum*. On the other hand, both TF and TPC in teas containing *L. casei* significantly reduced during the storage, whereas, in general, the TPC and AC of teas with *L. plantarum* remained constant during the storage. Based on these results, white tea and *L. plantarum* were used for further studies on bead production and gastrointestinal simulation.

**Table 1:** Probiotic count, total phenolic compounds, total flavonoids, and antioxidant capacity during the storage at room temperature (20 ± 3 °C) of black, green, and white teas

Time (days)	Probiotics (log10 CFU/g)					
	*L. casei*			*L. plantarum*		
	Black tea	Green tea	White tea	Black tea	Green tea	White tea
0	5.49 ± 0.48^a^	5.47 ± 0.46^a^	5.42 ± 0.48^a^	6.28 ± 0.27^a^	6.18 ± 0.68^a^	6.28 ± 0.25^a^
1	5.42 ± 0.36^a^	5.51 ± 0.32^a^	5.48 ± 0.16^a^	6.29 ± 0.39^a^	6.19 ± 0.35^a^	6.25 ± 0.42^a^
2	5.27 ± 0.45^a^	5.41 ± 0.45^a^	5.36 ± 0.52^a^	6.05 ± 0.53^a^	6.18 ± 0.67^a^	6.07 ± 0.44^a^
4	4.38 ± 0.32^b^	4.94 ± 0.25^a^	4.08 ± 0.85^b^	6.10 ± 0.43^a^	6.12 ± 0.44^a^	6.13 ± 0.32^a^

Time (days)	Total phenolic compounds (mg GAE/100 mL)					
	*L. casei*			*L. plantarum*		
	Black tea	Green tea	White tea	Black tea	Green tea	White tea
0	5.81 ± 0.02^a^	6.47 ± 0.02^a^	8.69 ± 0.01^a^	5.64 ± 0.01^a^	6.28 ± 0.03^a^	8.62 ± 0.02^a^
1	5.70 ± 0.02^b^	6.34 ± 0.00^b^	8.63 ± 0.03^b^	5.58 ± 0.05^a^	6.25 ± 0.01^a^	8.53 ± 0.02^b^
2	5.60 ± 0.05^b^	6.31 ± 0.01^b^	8.48 ± 0.03^c^	5.61 ± 0.04^a^	6.24 ± 0.02^ab^	8.64 ± 0.01^a^
4	5.34 ± 0.02^c^	6.43 ± 0.13^ab^	8.19 ± 0.01^d^	5.57 ± 0.08^a^	6.20 ± 0.01^b^	8.67 ± 0.05^a^

Time (days)	Total flavonoids (mg catechin/100 mL)					
	*L. casei*			*L. plantarum*		
	Black tea	Green tea	White tea	Black tea	Green tea	White tea
0	2.23 ± 0.03^a^	1.33 ± 0.02^a^	2.17 ± 0.10^a^	1.86 ± 0.08^b^	0.85 ± 0.04^c^	1.25 ± 0.01^c^
1	2.19 ± 0.10^a^	0.91 ± 0.04^c^	2.27 ± 0.08^a^	1.85 ± 0.09^b^	1.20 ± 0.06^b^	2.08 ± 0.09^b^
2	2.44 ± 0.20^a^	1.25 ± 0.05^a^	2.32 ± 0.06^a^	1.97 ± 0.02^ab^	0.91 ± 0.00^c^	2.38 ± 0.08^a^
4	1.71 ± 0.11^b^	1.11 ± 0.07^b^	1.38 ± 0.05^b^	2.02 ± 0.02^a^	1.44 ± 0.05^a^	2.06 ± 0.03^b^

Time (days)	Antioxidant capacity (mg Trolox/100 mL)					
	*L. casei*			*L. plantarum*		
	Black tea	Green tea	White tea	Black tea	Green tea	White tea
0	44.43 ± 2.32^b^	65.11 ± 1.10^a^	102.78 ± 6.16^b^	42.80 ± 0.76^b^	58.09 ± 2.93^a^	98.43 ± 6.16^bc^
1	50.71 ± 10.08^ab^	67.90 ± 1.10^a^	102.66 ± 1.16^b^	43.58 ± 2.07^b^	42.59 ± 0.40^b^	101.84 ± 1.16^b^
2	67.02 ± 12.60^a^	69.27 ± 1.19^a^	122.11 ± 1.73^a^	58.11 ± 2.98^a^	40.56 ± 1.45^b^	120.8 ± 1.73^a^
4	71.42 ± 16.50^a^	66.63 ± 0.87^a^	92.37 ± 2.44^b^	59.75 ± 0.66^a^	54.99 ± 5.14^a^	94.10 ± 2.44^c^

Average (*n* = 3) ± standard deviation. Different letters within the same column are statistically different

## Impact of gastrointestinal simulation on white tea beads with sodium alginate at different concentrations

**Fig. 4:**
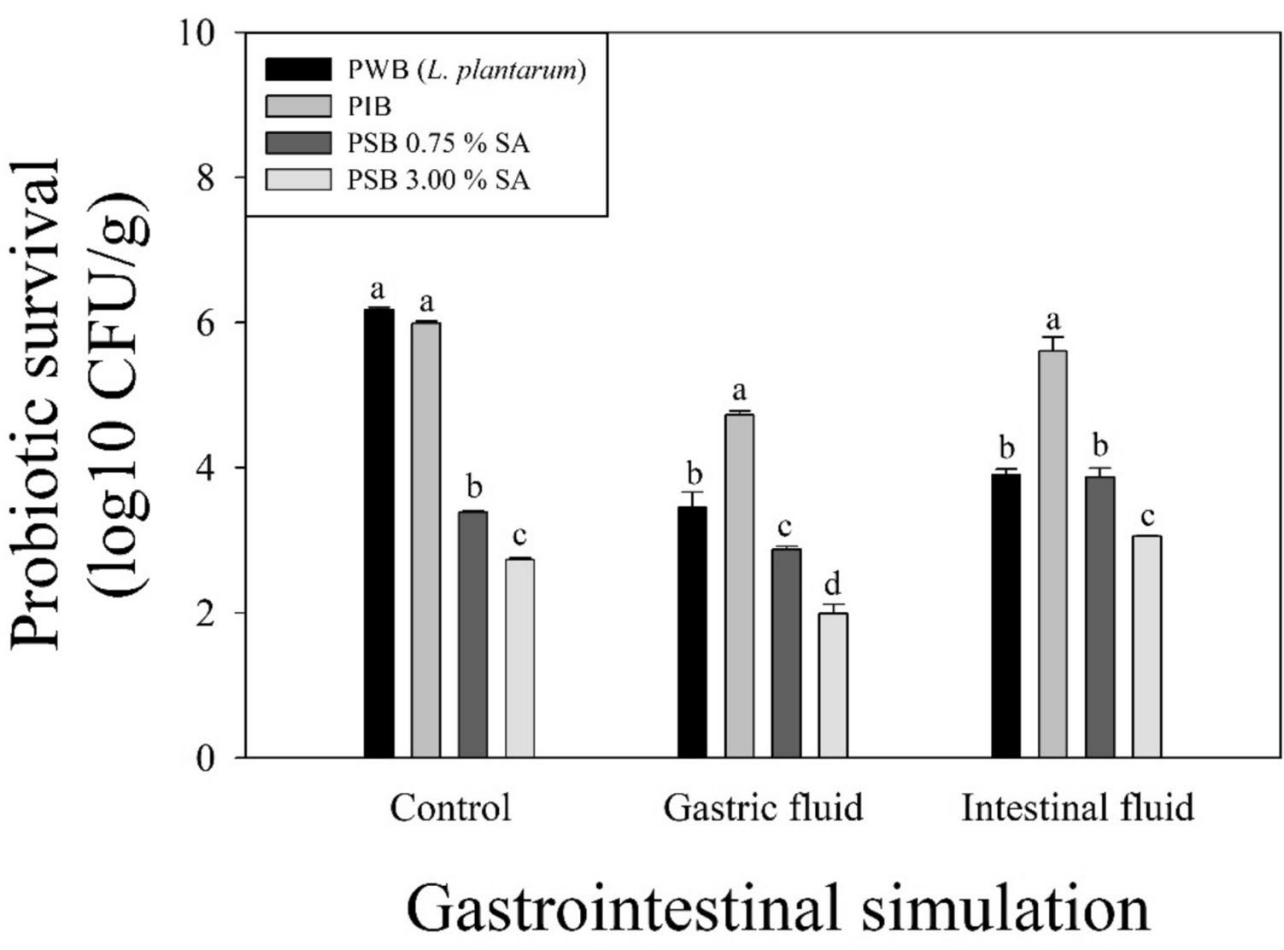
Gastrointestinal simulation of probiotic survival in white tea beads with sodium alginate. PWB: probiotic in the whole bead (PIB plus PSB). PIB: Probiotic inside the bead. PSB: probiotic on the surface of the bead at 0.75% and 3.00% sodium alginate. Different letters indicate statistical differences (p < 0.05). Bars indicate standard deviation

The effect of GS on inoculated white tea (control), the surface of the inoculated white tea bead (PSB), using either 0.75 or 3.00% SA, and probiotic in whole bead (PWB) is shown in Fig. 4. As observed, bead production did not affect the probiotic count (p > 0.05), which remained within the range of 5.99 ± 0.03 log10 CFU/g, compared with the inoculated white tea. On the other hand, at the surface of the beads (PSB), a significant effect (p < 0.05) on the survival of probiotics was observed with the concentration of SA in the bead formulation, decreasing from 3.39 ± 0.02 log10 CFU/g to 2.73 ± 0.02 log10 CFU/g when 0.75 and 3.00% of SA were used, respectively. This phenomenon can be attributed to crosslinking between alginate hydrogel and calcium ions, which prevents the probiotics from detaching from the bead surface (Yuan et al., 2022).

During the GS, probiotics without protection (control) were significantly affected by the gastric solution caused by its low pH, resulting in a 2.73 log10 reduction. In contrast, probiotics in beads showed a higher count after exposure to both gastric and intestinal fluids. However, when the analysis focused on the surface of the beads, which is the area where probiotics are liberated into the gastrointestinal system, the probiotic counts in beads formulated with 0. 75% SA decreased to 2.87 log10 CFU/g, while beads formulated with 3.00% SA exhibited even lower (p < 0.05) counts (1.99 ± 0.12 log10 CFU/g), likely due to the previously mentioned phenomenon. It is expected that, as probiotics are more effectively trapped within the beads (resulting in lower release into the fluids), the total number of probiotics remaining in the beads would be higher. These findings are consistent with those reported by Mandal et al. (2006), who noted that a higher probiotic concentration is retained in the bead when the SA concentration is increased in microencapsulated systems containing *L. casei*.

On the other hand, during the intestinal simulation, recovery is observed across all systems, a phenomenon reported in various studies on probiotics submitted to the GS. This recovery is probably due to the probiotic's ability to grow after stress induced by gastric fluid. In this regard, Stasiak-Różańska et al. (2021) reported that *L. plantarum* can recover as the pH of the gastrointestinal tract increases, which is crucial for its survival as it reaches the intestinal epithelium. Based on the results obtained, beads formulated with 0.75% SA were selected for further studies.

## Survival of probiotics on various parts of the bead under gastrointestinal simulation

**Fig. 5:**
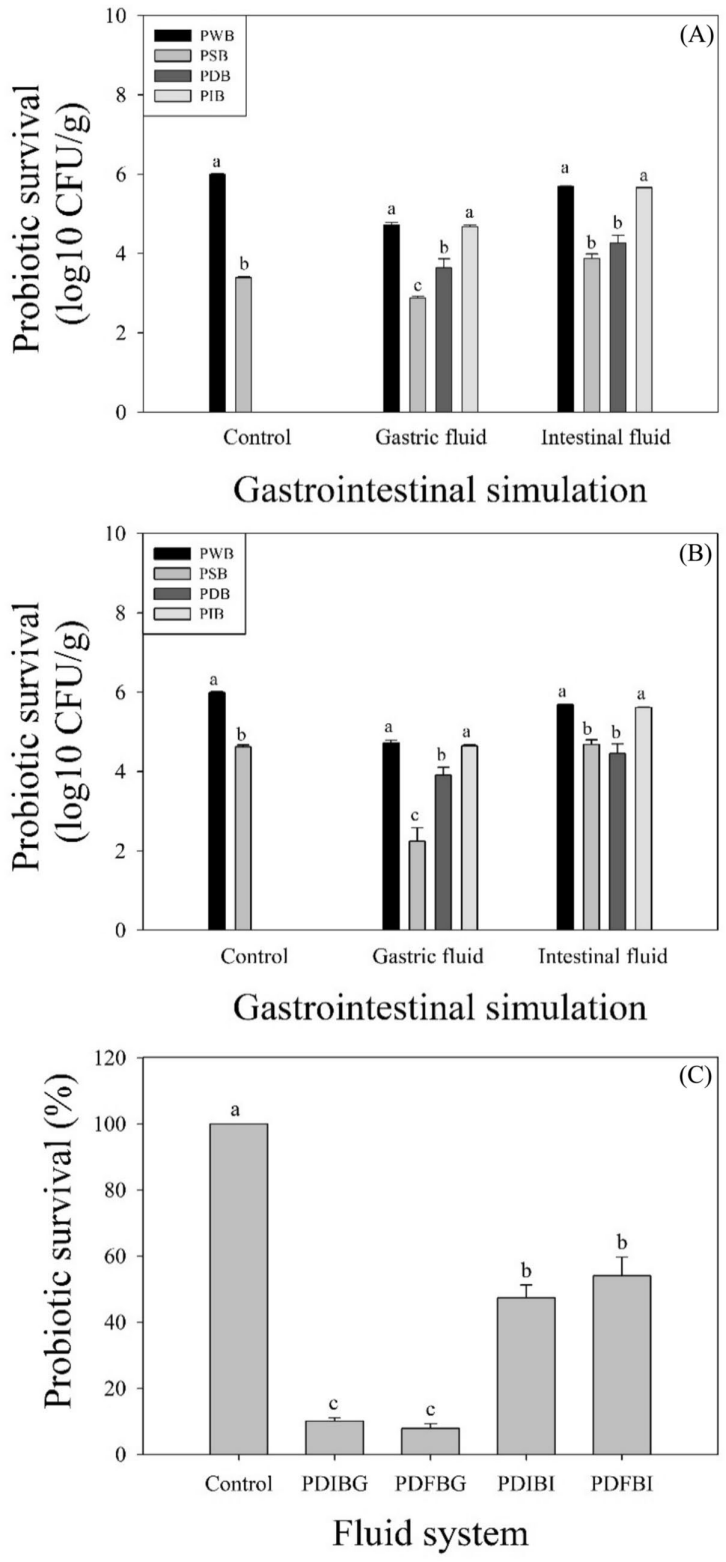
Probiotic survival on different parts of the bead and its surroundings, intact (**A**) and fractured (**B**) beads. Percentage of probiotic survival (**C**) in gastric (PDIBG and PDFBG) and intestinal fluids (PDIBI and PDFBI). PWB: probiotic in the whole bead (PIB plus PSB). PSB: probiotic on the surface of the bead. PDB: probiotic detached from the bead. PIB: probiotic inside the bead. Different letters indicate statistical differences (p < 0.05) among samples. Bars indicate standard deviation

This study hypothesizes that in the gastrointestinal system, probiotics can be in three different zones: 1) retained inside the bead (PIB) without being liberated into the gastrointestinal fluids, 2) on the surface of the bead (PSB), and 3) detached from it (PDB) to the surrounding gastric/intestinal fluid. The last two, PSB and PDB, could be liberated to the gastrointestinal system to exert their beneficial effect. Based on this hypothesis, and assuming that chewing can affect the release of probiotics from the bead, Fig. 5A and B show probiotic survival in different parts of the bead (0.75% sodium alginate) and its surrounding environments in intact and fractured beads, respectively. As mentioned before, during the gastrointestinal process, the probiotic count was significantly affected by gastric fluids; however, as shown in Fig. 5A, the surface of the bead (PSB) has the lowest probiotic count, followed by the detached probiotics in the gastric fluids (PDB). In contrast, the highest probiotic count was retained inside the bead (PIB), without being released into the gastrointestinal fluids. In a study conducted by Silva et al. (2022), the researchers evaluated the release of probiotics from capsules co-encapsulated with gelatin/gum Arabic and guaraná extracts. The findings indicated that during the gastric process, a higher number of probiotics (5—6 log10 CFU/g) were released into the gastrointestinal fluids compared to the results of this study, which showed 3.64—4.26 log10 CFU/g for intact beads and 3.91—4.46 log10 CFU/g for fractured beads. It's important to note that the probiotic count was significantly higher in the study conducted by Silva et al. (2022), ranging from 7.7 to 9.1 log CFU/g, while this study started with only 5.99 log10 CFU/g. This factor is crucial, as a higher initial probiotic count at the beginning of the gastrointestinal tract can lead to enhanced probiotic survival and potential health benefits for humans (Naissinger da Silva et al., 2021).

During the intestinal process, a recovery of probiotics was observed, with counts reaching 3.87 ± 0.13, 4.28 ± 0.20, and 5.66 ± 0.01 log10 CFU/g for the surface (PSB), fluids or detached (PDB), and retained (PIB) within the bead, respectively. The viability of probiotics (%) in gastric (PDIBG and PDFBG) and intestinal fluids (PDIBI and PDFBI) was also assessed using the MTT assay Fig. 5C. The results indicated that the fracturing process did not affect probiotic survival either in gastric or intestinal fluids. Furthermore, after the gastric process, the probiotics increased their survival in intestinal fluids, recovering from 47.36% to 54.10% during the intestinal process. This indicates that the fracturing process, which simulates chewing, does not impact the quantity or survival of probiotics (p > 0.05). Therefore, consumers can choose whether to chew the SA beads, as this will not compromise, at the simulation level, the survival of the probiotics.

Results indicated that white tea has the highest bioactive compounds and antioxidant capacity among the teas evaluated. On the other hand, between the *L. plantarum* and *L. casei*, the former probiotic showed the highest survival during storage. The beads formulated with white tea, *L. plantarum*, and the lowest amount of sodium alginate exhibit the highest probiotic liberation into gastrointestinal fluids. Furthermore, although the developed tea-based beads preserve a high population of probiotics during the gastrointestinal simulation, most of them were retained in the beads, while a smaller amount was released into the gastrointestinal fluids, which is of utmost importance since this portion is the one available to exert the benefits for human health.
